# Does integrated medical insurance system alleviate the difficulty of using cross-region health Care for the Migrant Parents in China-- evidence from the China migrants dynamic survey

**DOI:** 10.1186/s12913-021-07069-w

**Published:** 2021-10-05

**Authors:** Chao Ma, Shutong Huo, Hao Chen

**Affiliations:** 1 Southeast University, School of Economics and Management, Nanjing, Jiangsu China; 2 University of California, Irvine, Program of Public Health, Irvine, CA USA; 3 University of International Business and Economics, Institute of International Economy, Irvine, CA USA

## Abstract

**Background:**

Many internal migrants during the urbanization process in China are Migrant Parents, the aging group who move to urban areas to support their family involuntarily. They are more vulnerable economically and physically than the younger migrants. However, the fragmentation of rural and urban health insurance schemes divided by “hukou” household registration system limit migrant’s access to healthcare services in their resident location. Some counties have started to consolidate the Urban Resident Basic Medical Insurance (URBMI) and the New Rural Cooperative Medical Scheme (NRCMS) as one Integrated Medical Insurance Schemes (IMIS) from 2008. The consolidation aimed to reduce the disparity between different schemes and increase the health care utilization of migrants.

**Results:**

Using the inpatient sample of migrant parents from China Migrants Dynamic Survey in 2015, we used Ordinary Least Squares (OLS) for regression models. We found that the migrant parents covered by the IMIS are more likely to choose inpatient services and seek medical treatment in the migrant destination. We further subdivide Non-IMISs into NCMSs and URBMIs in the regression to alleviate the doubt about endogenous. The results revealed that the migrant parents in IMIS use more local medical services than both of them in URBMI and NCMS.

**Conclusions:**

The potential mechanisms of our results could be that IMIS alleviates the difficulty of seeking medical care in migrant destinations by improving the convenience of medical expense reimbursement and enhancing health insurance benefits.

## Introduction

The rapid economic growth of China has resulted in a historically unprecedented surge in urbanization. One of the critical reasons is that increasing numbers of rural inhabitants have joined this exodus to the cities in search of better job opportunities and improved quality of life. According to the seventh national census data in China in 2021 [1], the number of migrants reached 492.76 million. Compared with 2010, the population who lived in places other than their household registration areas went up by 88.52%. Another demographic characteristic of migrants in China is that more and more elderly members migrated with their families during the last two decades. The proportion of migrants aged over 45 years increased from 9.7% in 2010 to 12.9% in 2014 [2].

Among the large numbers of elderly migrants, the *Migrant Parents* (the aging groups who move to urban area involuntarily, and most of them move to support their family) is more vulnerable than others. This particular group is less economically and physically able to overcome the adverse effects of migration compared with younger migrants due to their physical, mental, and social network features. They are usually retired or unemployed with limited income and not eligible for health insurance for employees. However, China’s fragmented health insurance system caused a significant disparity between different residency statuses, which means the migrant aging groups from rural areas have difficulties fully enjoying health care in urban cities [3, 4]. Accordingly, the unmet health care utilization harms the *Migrant Parents’* well-being, as well as the health equity in Chinese society. This paper aims to discuss whether the integration of health insurance schemes could increase the health care utilization of *Migrant Parents* and satisfy their health needs. Our results also shed light on the universal health coverage worldwide, especially for the countries with national-level health insurance. We provide international evidence on how the national health insurance schemes could increase the health care utilization of vulnerable groups.

In fact, a large proportion of rural migrants in China are usually engaged in 3D (i.e., dirty, dangerous, and demanding) work that native residents are seldom willing to perform. They often work long hours at a higher intensity than native residents do, with less protection [5, 6]. However, the rural migrants are often systematically excluded from urban public resources due to the urban-rural residency, one of which is the access to healthcare [7, 8]. On the one hand, the Urban Employee Basic Medical Insurance (hereafter UEBMI) only covers the urban workers but excludes cover informal sector workers and migrant workers [9, 10]. On the other hand, the basic health insurance schemes used to be divided by “*hukou*”^1^ household registration system in China, Urban Resident Basic Medical Insurance (hereafter URBMI) for urban residents, and the New Rural Cooperative Medical Scheme (hereafter NCMS) for rural residents, causing considerable fragmentation in the health insurance system. Moreover, the identity-based schemes limit migrant’s access to healthcare services in their job locations because it is difficult for them to transfer the schemes from rural to urban and use a specific health insurance account across schemes [10].

Furthermore, URBMI and NCMS have independent administrative institution mechanisms and different financing pooling levels, which leads to low-risk protection ability and insufficient interconnections within the health insurance system, thereby causing significant inequity issues for migrants [11, 12]. In most regions, NCMS funds are pooled at the county level, while URBMI and UEBMI are pooled at the municipal (prefecture) level, implying that there are thousands of health insurance pools in China^2^ [4]. In this way, the reimbursement levels and benefits packages differ among schemes in different districts due to the disparity of economic development, which causes significant inequality in health care utilization between various schemes [4, 13]. Given that, migrants receive less coverage under formal medical schemes, and they encounter more barriers when applying for reimbursement of treatment expenses [14, 15].

As a result, the fragmentation of rural and urban health insurance schemes has been recognized as one of the most critical factors determining the disparities in social and economic development in China [16]. Thus, some provinces started to consolidate the NCMS and URBMIS as one Integrated Medical Insurance Schemes (hereafter IMIS). Meanwhile, the development of technology also makes it possible to realize real-time reimbursement across different regions. Therefore, the consolidation aims to raise the insurance pooling level, simplify the reimbursement process across regions and equalize the benefits package and risk protection ability among all groups of people [17].

Pushing even further, there are several reasons for us to care more about the consequence of IMIS on the *Migrant Parents* population. Although migrant workers face a higher risk of poor health and lower chances of accessing and affording treatment in cities, most studies found that migrants exhibit better health than natives because young and healthy individuals have a higher propensity to migrate [18–20]. Also, severe and incapacitating diseases and intensive-care conditions can result in a migrant’s return home to avoid the high medical and living costs in cities [21–23]. Instead of searching for job opportunities as young people do, the *Migrant Parents* group makes the decision based on family factors, like looking after their grandchildren [24]. Thus, the *Migrant Parents* group is more vulnerable than the young migrants to be affected by the inconvenience of reaching medical needs. Therefore, the integration of NCMS and URBMIS should benefit the *Migrant Parents* even more by increasing access to health care in their migrate destinations.

In addition, that *Migrant Parents* do have a higher prevalence and incidence of many diseases, especially chronic diseases. They need more health care services than the younger population due to the decline of resistance and physical function because of their aging [25–28]. Moreover, the *Migrant Parents* need to acculturate to a new environment and leave a familiar culture behind [29, 30]. The elders who used to live in villages lost their daily work, lifestyle, and community networks when their change residence from rural to urban [31]. This changing social environment has been linked to elder depression [32–35].

Consequently, we investigate in this study of great importance both in reality and in the literature. Using China Migrants Dynamic Survey, we found that the migrant parents covered by the IMIS are more likely to choose inpatient service and seek medical treatment in the migrant destination by improving the convenience of medical expense reimbursement and enhancing health insurance benefits. Thus, the potential mechanisms could be that IMIS alleviates the difficulty of seeking medical care in migrant destinations by improving the convenience of medical expense reimbursement and enhancing health insurance benefits. The remainder of the paper is organized as follows. First, we review some of the related empirical literature. Next, we describe the data and measurements and lays out the analytic strategy. Then we present our main findings. Finally, we discuss the implication of our results, the limitations of our work, and potential future directions.

## Literature review

### Health and health care utilization of the elderly in China

Illness increases with age, like cardiovascular disease, hypertension, cancer, osteoarthritis, et al. [25–28]. Like in China, the health of the elderly worsens with age, suffering from both cognitive and physical health issues [36]. In the case of China, the urban-rural dualistic structure has created a dual lifestyle and cultural belief [31]. As older people move, they face drastic changes in lifestyle and living environment, which World Health Organization has reported as the main factors affecting health. As a result, older people, especially migrant parents, have a more significant need for health care [37–39].

One of the most effective ways to satisfy the health care utilization among the elderly who need the services is to cover them the health insurance. A large body of literature shows that health insurance coverage can sharply increase health care utilization among elders [40–42]. For example, a study based on Medicare in the U.S. reveals that the universal insurance coverage increases the use of health care utilization among the elders [40]. Taiwan’s National Health Insurance coverage has also risen outpatient and inpatient care utilization among the elderly, and such effects were more salient for people in the low or middle-income groups [43].

In terms of China, increased health insurance coverage was accompanied by increased use of health care among the elderly [44]. The URBMI program has significantly increased the utilization of formal medical services, improving even more for the elderly [45]. It also has been shown that NCMS has improved the health care utilization of rural elders [46, 47]. However, URBMI has a more significant impact than other insurance policies since it receives more government finance than other schemes [48]. Liu and Wong found that the recruitment of URBMI increases the health care utilization, but signing up for NCMS does not improve both the utilization and health outcome among the elders [48]. However, rare researches are focusing on how IMIS influences health care utilization among elders.

### Health care utilization of the migrants

The migrant workers have made a tremendous contribution to China’s economic development. However, migrants face barriers to access to health care. Gong et al. suggested that migrant workers consistently underused health services both at their hometowns with *hukou* and residences [7]. In fact, rural-to-urban migrants are always excluded from city health systems because they cannot qualify for the UEBMI and URBMI as local city residents can, even when they are working in the same company and living in the same area community [49]. Even the employed migrants in urban areas are supposed to be covered by health insurance provided by their employers under UEBMI, employers usually lack motivation or pressure to do so [45]. As a result, they can only participate in their local NCMS, which in turn poses barriers when migrants seek health care in their destination cities.

Works of literature found that migrants have less healthcare utilization than their counterparts with urban residency [3, 50, 51]. In addition, people have made many complaints because of the poor portability of the schemes across locations, unsatisfactory transferability across the schemes, and weak interconnections among and within the schemes [11]. Specifically, seeking hospital care in out-of-county hospitals resulted in much lower reimbursement rates or even no reimbursement from the NCMS [52], which might lead to lower healthcare utilization than they actually need. In terms of the aging groups in migrants, according to the 2015 China Migrants Dynamic Survey (CMDS), 54.27% of the elderly migrants preferred being either self-treated or untreated rather than visiting hospitals; 18% requiring hospitalization did not use the inpatient service. Among those who received the inpatient service, 30% returned to their hometown for hospitalization [53].

### Chinese basic health insurance system and the integrated medical insurance schemes

China has spent a long time on health insurance reform and successfully achieved universal health insurance coverage in 2011, by which 95% of the Chinese population was insured compared with less than 50% in 2005 [54]. The coverage was offered through public insurance programs in China, New Rural Cooperative Medical Scheme (NCMS), Urban Resident Basic Medical Insurance (URBMI), and Urban Employee Basic Medical Insurance (UEBMI). In 2003, China launched NCMS, a significantly subsidized voluntary health insurance program for rural residents. It serves as a replacement for the old village-based rural health insurance program. Most rural-urban migrants were enrolled in the NCMS due to their residency. On the other hand, URBMI started in July 2007, providing coverage for the urban residents without formal jobs or unemployed such as children, students, the elderly, and the young unemployed. While NCMS and URBMI cover most residents in rural and residents without a job in urban, UEBMI aims to provide health insurance to employed urban residents. Based on the pilot reforms in the cities of Zhenjiang and Jiujiang, UEBMI was proposed to replace the government insurance scheme and the labor insurance scheme [55, 56]. In general, UEBMI stipulates that the employment-based basic health insurance scheme should cover urban employees, including workers from both public and private enterprises. Retired workers are exempted from premium contributions, and their former employers should shoulder the costs of their contributions. It means that the elderly migrants who did not retire in urban areas are not beneficiaries of UEBMI.

The fragmentation of rural and urban health insurance systems was characterized as a determinant of the disparities in social and economic development in China [16]. As a result, the integration of NCMS and URBMI was an urgent need. Since 2008, some provinces and cities have started to practice the consolidation of two residential insurance schemes. However, the reform in the pilot area was not sufficient due to the absence of institutional design and guidelines from the national government [57–59]. To move forward to the thorough reform, in 2015, the leader in China announced the decision to merge the NCMS and URBMI. In January 2016, China officially issued a document on integrating NCMS and URBMI regarding insurance coverage, funding policies, insured treatment, reimbursement catalogs, contracted medical institutions, and fund management called Integrated Medical Insurance Schemes (IMIS) [60]. Furthermore, aiming to break the limitation of fragmented administration, the National Healthcare Security Administration was launched in March 2018, which oversees and manages the health insurance plan, drug price, purchase, medical aid, and maternity insurance at the national level [61].

In 2019, 24 provinces had integrated the NCMS and URBMI and operated the IMIS. A document from the National Healthcare Security Administration has emphasized that the rest seven provinces should increase the consolidating schemes process [62].

## Methods

### Data collection

We use the 2015 China Migrants Dynamic Survey (CMDS) in this study, conducted by the National Population and Family Planning Commission.^3^ The survey covers all 32 provinces of China, 348 cities, and 10,300 communities or villages. The 2015 CMDS adopted a stratified three-stage probability proportionate to size (PPS) sampling, and the annual national data on migrants from each province in 2014 was considered the basic sampling frame. In each selected community, they chose 20 eligible individual migrants randomly to participate in the survey. The migrant participants of the household survey are between 16 and 59 years old and have moved across a county boundary from their registered household and lived in a city for more than one month. This round also included the migrants aged over 60 with information about the household, employment, and healthcare. The sample is representative at the national and provincial levels.

### Model

To examine the relationship between health care utilization and the IMIS policy, we use the following model:

1 $$ {HC}_{ij}=\beta {imis}_{ij}+\gamma need\_{inpa}_{ij}+\varnothing {X}_{ij}+{region}_j+{\varepsilon}_{ij} $$

Where *HC*_*ij*_ is our dependent variable, health care utilization of individual *i* in region *j*. The key independent variable in this study was *imis*_*ij*_ (whether the individual participated in IMIS), it equals one if the person participates in IMIS and 0 if otherwise. *need* _ *inpa*_*ij*_ denotes people have had an illness/injury diagnosed by doctors in the past year that requires hospitalization. This model also includes a set of control variables *X*_*ij*_. *region*_*j*_ represents the fixed effects of the original provinces as well as the flow-in cities. *ε*_*ij*_ is the error term.

There are two main indexes in this study: hospitalized in the past year (*inpa*) and hospitalized locally in the past year (*local_inpa*). If a doctor determines that the patient needs to be hospitalized, whether patients choose to be hospitalized and whether they choose to be hospitalized locally is a good indicator of the accessibility of medical services. Specifically, suppose we control the variable *need* _ *inpa* which can reflect the needs of hospitalization. In that case, *inpa* can effectively represent proper health care or not has been received by people. Furthermore, it is helpful for us to investigate the impact of an integrated medical insurance system (IMIS) on alleviating the difficulty of *Migrants Parents* (parents who are driven to follow their children to other cities) to seek medical treatment. The other variable *local_inpa* can effectively identify whether an individual is enjoying local medical resources or not treating the sick locally, just like going back home for treatment. We also have another dependent variable *less_serious_doctor* (whether people will see the doctor locally if they get a less severe disease) for reference only, reflecting whether *Migrants Parents* have a problem with excessive medical care.

Specifically, the series of control variables *X*_*ij*_ include: household incomes per capita, household expenditure per capita, household expenditure on food per capita, household expenditure on the house per capita, *hukou* status, education level, and the principal source of income are used to evaluate the socioeconomic status (SES) of the individuals. Furthermore, whether diagnosed with diabetes or hypertension, self-reported health status, and whether has the inpatient need are the health status indicators. In addition, we controlled for the fitness time per week, and whether has the medical examination this year as the proxy of the health behavior variables. Additionally, we controlled gender, age, ethnicity, and marital status as the demographic variables. Finally, we added the number of friends in the flow-in cities, years since migration (YSM), and the main reason for migration as the proxy of the migration status. Table 1 reports descriptive statistics for the main variables.

**Table 1:** Descriptive Statistics of Main Variables by Types of Health Insurance Schemes

Variable	Non-IMISs		IMISs		NCMSs		URBMIs	
	Mean	Std. Dev.	Mean	Std. Dev.	Mean	Std. Dev.	Mean	Std. Dev.
*hospitalized in the past year (inpa)*	0.080	0.272	0.096	0.295	0.082	0.275	0.069	0.254**
*hospitalized locally in the past year (local_inpa)*	0.058**	0.234	0.081	0.274	0.059**	0.235	0.056	0.231**
*see a doctor locally with less serious diseases (less_serious_doctor)*	0.451	0.498	0.459	0.499	0.447	0.497	0.475	0.500
*the need of hospitalization (need_inpa)*	0.098	0.298	0.108	0.311	0.100	0.300	0.089	0.285
self-reported health status	3.313	0.722	3.355	0.718	3.306*	0.725	3.356	0.703
hypertension or diabetes (1 = have, 0 = no)	0.217	0.412	0.211	0.408	0.212	0.408	0.250	0.433*
age	66.544	6.140	66.690	6.293	66.491	6.152	66.861	6.058
gender (1 = male, 0 = female)	0.512	0.500	0.506	0.500	0.518	0.500	0.469	0.499
marriage (1 = yes, 0 = no)	0.794**	0.405	0.827	0.379	0.784**	0.412	0.850	0.357
ethnic (1 = han, 0 = minority)	0.901***	0.298	0.943	0.232	0.898***	0.302	0.918	0.274*
*hukou* (1 = rural, 0 = urban)	0.852***	0.355	0.569	0.496	0.951***	0.216	0.256	0.436***
household incomes per capita per month	1918.866	11,869.640	2091.701	1984.529	1865.950	12,790.580	2238.668	1826.181
household expenditure per capita per month	959.597***	714.009	1109.474	780.363	917.887***	677.181	1211.721	863.547**
household food expenditure per capita per month	423.231***	281.020	496.829	342.393	404.087***	265.220	539.101	340.100**
household house expenditure per capita per month	179.260***	298.014	231.996	290.534	173.898***	280.378	211.512	386.469
Number of friends in residence	7.486***	9.401	8.562	8.884	7.170***	8.951	9.390	11.585
years since migration	6.674***	6.704	5.380	5.206	6.532***	6.505	7.523	7.747***
fitness_time per day (min)	62.302***	45.562	73.432	48.234	60.413***	45.259	73.694	45.732
Health examination in the past one year	0.315***	0.465	0.447	0.498	0.301***	0.459	0.400	0.490*
Main source of income								
Self-employment(1 = yes, 0 = no)	0.279***	0.448	0.232	0.422	0.296***	0.456	0.176	0.381***
Pension and Savings(1 = yes, 0 = no)	0.216***	0.412	0.387	0.487	0.158***	0.364	0.569	0.495***
Support from other family numbers(1 = yes, 0 = no)	0.428***	0.495	0.312	0.464	0.466***	0.499	0.198	0.399***
Others(1 = yes, 0 = no)	0.077	0.266	0.069	0.254	0.080	0.272	0.056	0.231
N	7250		664		6222		1029	

*Note*: * *p* < 0.1, ** *p* < 0.05, *** *p* < 0.01, representing statistical significance compared with IMISs. Non-IMISs, IMISs, NCMSs, URBMIs represent Migrants Parents who have not joined IMIS and who joined IMIS, NCMS and URBMI, while Non-IMISs is the combination of NCMSs and URBMIs. Self-reported health status include no self-care ability, poor but with self-care ability, fair and good, ranked from 1 to 4; Education includes no formal education, elementary school, middle school, high school/vocational school, and college and above, ranked from 1 to 5. Self-reported health status is shown as continuous variable in this table. The main results are robust when considering health status as dummy or continuous variable in the following regressions

## Results

### Descriptive statistics

The descriptive statistics in Table 1 can partially explain the issues concerned in this paper. By comparing *Migrants Parents* who have already joined IMIS (we define them as IMISs) and who have not (non-IMISs), we find that when the mean value of *need_inpa* was almost the same (0.108 and 0.098), the mean value of IMISs on *local_inpa* was much higher than that of non-IMISs (0.081 and 0.058). It means that when *Migrants Parents* are deemed to be in the hospital by the doctor, IMISs will choose to be in hospital locally and enjoy local medical services instead of those in their hometown. We emphasize the mean value of *need_inpa* is almost the same because the proportion of *Migrants Parents* who need to be hospitalized due to illness must be guaranteed to be similar. As a result, it is meaningful to compare the proportion of local hospitalization of IMISs and Non-IMISs, which can reflect the improvement in the utilization efficiency of local medical services with the aid of IMIS. In addition, there is also a difference in *inpa* between IMISs and Non-IMISs (0.096 and 0.080), which proves that if hospitalization is indeed required, IMISs will be less likely to “not go to treatment for illness.”

Other variables also show interesting patterns. We find that IMISs have higher SES than non-IMISs, including higher education (2.312 and 2.135), more non-agricultural *hukou* (0.569 and 0.852), higher monthly household income per capita (2091.701 and 1918.866), and expenditure per capita (1109.474 and 959.597). Also, we find IMISs have more local friends (8.562 and 7.486), adequate exercise time (73.432 and 62.302), and more regular physical examination (0.447 and 0.315). These statistical results have two meanings: on the one hand, it indicates that many factors influence the results, which need to be controlled in the following regression. On the other hand, it implies a possible risk of selection bias. For example, those IMISs who seem to be more locally hospitalized and hospitalized have higher SES, instead of that IMIS is the main reason why they chose to be inpatient service locally. In other words, it is possible that the area that implements IMIS might have better economic status than those without IMIS. To eliminate this doubt, non-IMISs are further divided into NCMSs (*Migrants Parents* who only participate in the New Rural Cooperative Medical System) and URBMIs (*Migrants Parents* who only participate in the Urban Residents Basic Medical Insurance). Because it can be seen that although the SES of IMISs is significantly higher than that of NCMSs, it is not entirely higher than that of URBMIs. IMISs are even lower than URBMIs in years of education, marital status, monthly household income and expenditure per capita, number of local friends, and average daily exercise time. Even so, IMISs were still higher than URBMIs in the mean value of the two dependent variables, and the selection bias has little influence on the results. Higher SES cannot fully explain the improvement in the efficiency of enjoying local medical resources. The *Migrants Parents* who need to be hospitalized choose to go to hospital and stay in local hospitals more often should be attributed to IMIS policy.

### Benchmark results

Table 2 reports the benchmark results,^4^ whose dependent variables are *inpa*, *local_inpa,* and less_serious_doctor. Columns (1)–(3) of Table 2 focus on IMISs vs. Non-IMISs, columns (4)–(6) focus on IMISs vs. NCMSs, and columns (7)–(9) focus on IMISs vs. URBMIs. Columns (1), (4), and (7) control the health status, health behaviors, individual demographic characteristics, and city fixed effect besides *imis*. Columns (2), (5), and (8) also control SES and immigration information based on (1), (4), and (7). Columns (3), (6), and (9) also control *need_inpa* based on (2), (5), and (8). Table 3 has the same structure as Table 2. The regression results in Table 4 are for reference only because the dependent variable is not a fact but a subjective attitude, *Migrants Parents’* willingness to seek medical treatment locally even if they are just a little ill. At the same time, we realize that the effect of IMIS is less obvious compared with severe diseases requiring hospitalization, which is shown in the weaker coefficient of *imis* in Table 4. It is also intuitive: for minor illnesses, the requirements for reimbursement, price, and medical convenience are lower. Many *Migrants Parents* are still willing to go to local hospitals even without medical insurance.

**Table 2:** Comparison of Health Care Utilization of Migrant Parents in Residency between IMISs and non-IMISs

	(1)	(2)	(3)	(4)	(5)	(6)	(7)	(8)
	*hospitalized in the past year(inpa)*	*hospitalized in the past year(inpa)*	*hospitalized in the past year(inpa)*	*hospitalized locally in the past year(local_inpa)*	*hospitalized locally in the past year(local_inpa)*	*hospitalized locally in the past year(local_inpa)*	*see a doctor locally with less serious diseases(less_serious_doctor)*	*see a doctor locally with less serious diseases(less_serious_doctor)*
*imis*	0.0315^**^	0.0315^**^	0.0163^**^	0.0330^***^	0.0337^***^	0.0226^***^	0.0446^*^	0.0401
	(0.0128)	(0.0127)	(0.00749)	(0.0121)	(0.0119)	(0.00809)	(0.0269)	(0.0250)
*the need of hospitalization (need_inpa)*			0.818^***^			0.594^***^		
			(0.0173)			(0.0242)		
self-reported health status	−0.0811^***^	−0.0735^***^	− 0.00194	− 0.0632^***^	− 0.0575^***^	− 0.00558	0.00617	0.00873
	(0.00713)	(0.00748)	(0.00307)	(0.00655)	(0.00681)	(0.00430)	(0.0110)	(0.0109)
Having hypertension or diabetes	0.100^***^	0.0958^***^	0.00470	0.0854^***^	0.0823^***^	0.0162^**^	0.0347^**^	0.0335^**^
	(0.0115)	(0.0112)	(0.00410)	(0.0110)	(0.0108)	(0.00645)	(0.0151)	(0.0148)
Fitness time per day (min)	0.000134	0.0000815	0.0000116	0.0000971	0.0000489	−0.00000183	0.000351^**^	0.000181
	(0.0000876)	(0.0000852)	(0.0000343)	(0.0000786)	(0.0000769)	(0.0000434)	(0.000156)	(0.000153)
Health examination in the past one year	−0.00183	− 0.00293	− 0.00360	0.00305	0.00182	0.00134	0.0755^***^	0.0715^***^
	(0.00821)	(0.00811)	(0.00357)	(0.00803)	(0.00788)	(0.00461)	(0.0204)	(0.0205)
age	0.00153^**^	0.000946	0.000113	0.00113^*^	0.000631	0.0000274	0.00488^***^	0.00321^**^
	(0.000691)	(0.000761)	(0.000271)	(0.000630)	(0.000689)	(0.000392)	(0.00122)	(0.00127)
male	0.00793	0.0126^**^	0.00258	0.00547	0.00823^*^	0.000999	−0.0365^***^	−0.0236^**^
(reference group: female)	(0.00562)	(0.00587)	(0.00280)	(0.00491)	(0.00497)	(0.00345)	(0.00845)	(0.00950)
Married	0.00237	0.000302	0.000569	−0.00316	−0.00437	−0.00418	− 0.0171	−0.0107
(reference group: Unmarried)	(0.00887)	(0.00873)	(0.00415)	(0.00767)	(0.00758)	(0.00516)	(0.0161)	(0.0167)
Han ethnic	−0.000451	0.00125	0.00373	−0.00464	−0.00464	− 0.00285	−0.0280	− 0.0322
(reference group: minority)	(0.0127)	(0.0127)	(0.00752)	(0.0115)	(0.0116)	(0.0100)	(0.0281)	(0.0273)
Elementary school		0.00266	−0.00218		−0.00233	−0.00584		−0.0170
(reference group: no formal education)		(0.00986)	(0.00443)		(0.00790)	(0.00499)		(0.0178)
Middle school		−0.0157	−0.0102^**^		−0.0127	−0.00867		0.000487
(reference group: no formal education)		(0.0118)	(0.00515)		(0.00988)	(0.00574)		(0.0213)
High school/vocational school		0.000738	−0.00193		0.00700	0.00507		−0.0170
(reference group: no formal education)		(0.0162)	(0.00682)		(0.0145)	(0.00799)		(0.0384)
college and above		−0.0709^***^	−0.0225^*^		−0.0621^***^	−0.0270^*^		−0.0617
(reference group: no formal education)		(0.0187)	(0.0136)		(0.0171)	(0.0145)		(0.0604)
rural		0.0101	0.000679		0.00742	0.000608		−0.0321
(reference group: urban)		(0.00892)	(0.00527)		(0.00799)	(0.00585)		(0.0206)
income		−0.00474	−0.000639		0.00472	0.00770^**^		0.0275^**^
		(0.00521)	(0.00211)		(0.00319)	(0.00327)		(0.0120)
expenditure		0.0166	−0.000869		0.00600	− 0.00663		− 0.0205
		(0.0108)	(0.00423)		(0.00788)	(0.00527)		(0.0215)
Food expenditure		−0.00831	0.00225		−0.00677	0.000888		−0.000658
		(0.00773)	(0.00361)		(0.00669)	(0.00443)		(0.0155)
House expenditure		−0.000830	−0.000593		−0.000495	−0.000323		−0.00820^**^
		(0.00154)	(0.000736)		(0.00136)	(0.000929)		(0.00377)
Main source of income								
Pension and Savings		0.0279^**^	−0.0151^**^		0.0252^**^	−0.00598		0.0417
(reference group: self-employment)		(0.0137)	(0.00688)		(0.0120)	(0.00799)		(0.0298)
Support from other family numbers		0.0110	−0.0143^**^		0.0152	−0.00315		0.0585^**^
(reference group: self-employment)		(0.0131)	(0.00638)		(0.0123)	(0.00756)		(0.0280)
Others		0.0109	−0.0145^*^		0.00732	−0.0111		−0.0179
(reference group: self-employment)		(0.0183)	(0.00755)		(0.0163)	(0.00996)		(0.0336)
Number of friends in residence		0.000474	0.0000575		0.000582	0.000280		0.00251^***^
		(0.000450)	(0.000152)		(0.000388)	(0.000250)		(0.000959)
years since migration		0.000649	0.000408		0.00140^***^	0.00122^***^		−0.00107
		(0.000557)	(0.000302)		(0.000519)	(0.000366)		(0.00122)
Main source of income		0.0141	0.0242^***^		0.00457	0.0119		0.0198
		(0.0123)	(0.00773)		(0.0115)	(0.00791)		(0.0293)
Fixed effects of origin provinces	√	√	√	√	√	√	√	√
Fixed effects of flow-in cities	√	√	√	√	√	√	√	√
_cons	0.121^*^	0.0781	0.0588^*^	0.163^***^	0.114	0.0996^**^	0.591^***^	0.702^***^
	(0.0616)	(0.0835)	(0.0319)	(0.0589)	(0.0776)	(0.0486)	(0.122)	(0.175)
*N*	7919	7912	7912	7919	7912	7912	7919	7912

*Note*: Robust standard errors are reported in parentheses. * *p* < 0.1, ** *p* < 0.05, *** *p* < 0.01. The dependent variable of Columns (1)– (3) is inpa; the dependent variable of Columns (4)– (6) is local_inpa; the dependent variable of Columns (7)– (8) is less_serious_doctor. The Column (1) (4), and (7) only control health status, health behaviors, individual demographic characteristics and city fixed effect. The Column (2) (5), and (8) also controls SES, including education, income, expenditure and immigration information, on the basis of Column (1) (4), and (7). The Column (3) and (6) also controls need_inpa on the basis of Column (2) and (5). Since the dependent variable of Column (7) and (8) is less_serious_doctor (the circumstance that the Migrant Parents do not need inpatient services), we do not control need_inpa in these two columns

**Table 3:** Comparison of Health Care Utilization of Migrant Parents in Residency between IMISs and NCMSs

	(1)	(2)	(3)	(4)	(5)	(6)	(7)	(8)
	*hospitalized in the past year(inpa)*	*hospitalized in the past year(inpa)*	*hospitalized in the past year(inpa)*	*hospitalized locally in the past year(local_inpa)*	*hospitalized locally in the past year(local_inpa)*	*hospitalized locally in the past year(local_inpa)*	*see a doctor locally with less serious diseases(less_serious_doctor)*	*see a doctor locally with less serious diseases(less_serious_doctor)*
*imis*	0.0338^**^	0.0232^*^	0.0109^*^	0.0367^***^	0.0283^**^	0.0195^***^	0.0572^**^	0.0436
	(0.0139)	(0.0141)	(0.00646)	(0.0130)	(0.0129)	(0.00728)	(0.0287)	(0.0310)
*the need of hospitalization(need_inpa)*			√			√		
Other control variables	√	√	√	√	√	√	√	√
SES		√	√		√	√		√
Fixed effects of origin provinces	√	√	√	√	√	√	√	√
Fixed effects of flow-in cities	√	√	√	√	√	√	√	√
*N*	6891	6885	6885	6891	6885	6885	6891	6885

*Note*: Robust standard errors are reported in parentheses. * *p* < 0.1, ** *p* < 0.05, *** *p* < 0.01. Other control variables include individual demographic characteristic, self-reported health status, having hypertension or diabetes, fitness time, having health examination or not in the past year. SES includes education, income, expenditure and immigration information. Omit specific results. Same structure of dependent variable as in Table 2

**Table 4:** Comparison of Health Care Utilization of Migrant Parents in Residency between IMISs and. URBMIs

	(1)	(2)	(3)	(4)	(5)	(6)	(7)	(8)
	*hospitalized in the past year(inpa)*	*hospitalized in the past year(inpa)*	*hospitalized in the past year(inpa)*	*hospitalized locally in the past year(local_inpa)*	*hospitalized locally in the past year(local_inpa)*	*hospitalized locally in the past year(local_inpa)*	*see a doctor locally with less serious diseases(less_serious_doctor)*	*see a doctor locally with less serious diseases(less_serious_doctor)*
*imis*	0.0358^*^	0.0327	0.0172^**^	0.0341^*^	0.0322	0.0195^*^	0.0347	0.0228
	(0.0199)	(0.0200)	(0.00711)	(0.0201)	(0.0209)	(0.0109)	(0.0442)	(0.0525)
*the need of hospitalization(need_inpa)*			√			√		
Other control variables	√	√	√	√	√	√	√	√
SES		√	√		√	√		√
Fixed effects of origin provinces	√	√	√	√	√	√	√	√
Fixed effects of flow-in cities	√	√	√	√	√	√	√	√
*N*	1692	1691	1691	1692	1691	1691	1692	1691

*Note*: Robust standard errors are reported in parentheses. * *p* < 0.1, ** *p* < 0.05, *** *p* < 0.01. Other control variables include individual demographic characteristic, self-reported health status, having hypertension or diabetes, fitness time, having health examination or not in the past year. SES includes education, income, expenditure and immigration information. Omit specific results. Same structure of dependent variable as in Table 2

We need to focus on explaining the regression results in Tables 2 and 3. First of all, in columns (1)–(6) of Tables 2 and 3, the coefficient of *imis* is positive, showing that compared with Non-IMISs or NCMSs, migrant parents who are in IMIS enjoy more local medical services. Especially the coefficient of *imis* is significantly positive in column (3) and (6), which shows that it is considerably easier for IMISs to be hospitalized in local hospitals when they are sick and needs to be hospitalized. In columns (7)–(9) of regression comparing IMISs and URBMIs, the coefficient of *imis* is still significantly positive in most cases, but the significance level has decreased. It may be due to the small sample size of URBMIs, so it does not affect the establishment of the conclusions in this paper. Further comparison between Table 2 and Table 3 shows that when the dependent variable is *local_inpa*, the coefficient of *imis* is more significant and larger. It indicates that IMIS makes *Migrants Parents* more willing to stay in local hospitals when they need to, instead of going back to their hometown for hospitalization. It also reflects the medical convenience IMIS brings to migrant parents. It also reflects the medical convenience which IMIS brings to migrant parents.

In addition, we do not worry too much about endogenous even if we only use OLS for cross-section data. On the one hand, the self-selection mentioned above will be alleviated by further subdivision. On the other hand, Basic medical insurance in China is fixed on individuals by their *hukou* and local medical insurance policies, so there is little adverse selection of medical insurance by individuals. Moreover, the migration of migrant parents is often passive (they follow their children to a migrant), so there is almost no self-selection bias for *Migrants Parents*. In summary, OLS results based on cross-section data in our research are reliable.

## Robustness check

### Selection bias problem of IMIS policy

We further subdivide Non-IMISs into NCMSs and URBMIs in the regression to alleviate the doubt about endogenous. As we mentioned above, there is a significant disparity between NCMSs and URBMIs on SES, while the mean value of SES of IMISs is just between them. Therefore, we compare IMISs with URBMIs in Table 3 and with NCMSs in Table 4 separately. The results reveal that the migrant parents in IMIS use more local medical services than both of them in URBMI and NCMS. Therefore, we don’t need to worry that the positive influence of IMIS on the dependent variable comes from the self-selection of the SES dominant group (URBMIs).

### Adjust for possible IMIS misinformation and distortion

In our data used in this paper, there are 1029 URBMIs, among which 263 (25.56%) are rural residents with agriculture *hukou*. According to the policy in China, rural residents with agriculture *hukou* can only participate in NCMS, so it appears a paradox. The most likely fact is that these people are actually IMISs but misreport or join insurance types repeatedly. First, in most regions, IMIS is a process that is promoted from NCMS with lower reimbursement treatment to URBMI with higher reimbursement treatment. For the rural elderly, it is also a process of realizing the treatment of urban residents, making them mistakenly believe that they have become URBMIs. Second, in China, NCMS is usually administrated by the local health department, while URBMI is generally administrated by the local human resources and social security department. When NCMS and URBMI are merged into IMIS, IMIS will be administrated by the local human resources and social security department. In this way, it is easy for *Migrants Parents* with agricultural *hukou* to mistake themselves for URBMIs. Third, the difference between URBMI and IMIS is only one character in Mandarin Chinese. *Migrants Parents* with agricultural *hukou* often have a low level of education. Therefore, it is possible to mistake URBMI for IMIS when they answer questions in the questionnaire.

As a result, we conduct a robustness check in which the 263 samples are regarded as IMISs, and the results are shown in Table 5. In addition, we adjusted the repeated insurance enrollment in Table 6. Columns 1–3 in Tables 5 and 6 are equivalent to columns 3, 6, and 8 in Table 2. Columns 4–6 in Tables 5 and 6 are equal to columns 3, 6, and 8 in Table 3. Columns 7–9 in Tables 5 and 6 are equivalent to columns 3, 6, and 8 in Table 4. After the adjustment for the possible data deviation, the conclusion has not changed.

**Table 5:** Comparison of Health Care Utilization of Migrant Parents between IMISs and. URBMIs after the Adjustment of Insurance Type

	(1)	(2)	(3)	(4)	(5)	(6)	(7)	(8)	(9)
	IMISs vs. non-IMISs			IMISs vs. NCMSs			IMISs vs. URBMIs		
	*hospitalized in the past year(inpa)*	*hospitalized locally in the past year(local_inpa)*	*see a doctor locally with less serious diseases(less_serious_doctor)*	*hospitalized in the past year(inpa)*	*hospitalized locally in the past year(local_inpa)*	*see a doctor locally with less serious diseases(less_serious_doctor)*	*hospitalized in the past year(inpa)*	*hospitalized locally in the past year(local_inpa)*	*see a doctor locally with less serious diseases(less_serious_doctor)*
*imis*	0.0147^**^	0.0244^***^	0.0431^*^	0.0109^*^	0.0195^***^	0.0436	0.0321^***^	0.0228	0.0125
	(0.00596)	(0.00696)	(0.0227)	(0.00646)	(0.00728)	(0.0310)	(0.00968)	(0.0525)	(0.0561)
*the need of hospitalization(need_inpa)*	√	√		√	√		√	√	
Other control variables	√	√	√	√	√	√	√	√	√
SES	√	√	√	√	√	√	√	√	√
Fixed effects of origin provinces	√	√	√	√	√	√	√	√	√
Fixed effects of flow-in cities	√	√	√	√	√	√	√	√	√
*N*	7912	7912	7912	6885	6885	6885	1691	1691	1691

*Note*: Robust standard errors are reported in parentheses. * *p* < 0.1, ** *p* < 0.05, *** *p* < 0.01. Other control variables include individual demographic characteristic, self-reported health status, having hypertension or diabetes, fitness time, having health examination or not in the past year. SES includes education, income, expenditure and immigration information. Same structure of dependent variable as in Table 2. The columns (1) – (3) and (4)– (6) have same structure as column (3), (6), and (8) in Table 2. The columns (7)– (9) have same structure as column (3), (6), and (8) in Table 4. In this regression, we redefine the potentially misreported insurance type, considering samples who are rural residents with agriculture *hukou* as IMISs

**Table 6:** Comparison of Health Care Utilization of Migrant Parents between IMISs and. URBMIs after Dropping Repeated Enrollment

	(1)	(2)	(3)	(4)	(5)	(6)	(7)	(8)	(9)
	IMISs vs. non-IMISs			IMISs vs. NCMSs			IMISs vs. URBMIs		
	*hospitalized in the past year(inpa)*	*hospitalized locally in the past year(local_inpa)*	*see a doctor locally with less serious diseases(less_serious_doctor)*	*hospitalized in the past year(inpa)*	*hospitalized locally in the past year(local_inpa)*	*see a doctor locally with less serious diseases(less_serious_doctor)*	*hospitalized in the past year(inpa)*	*hospitalized locally in the past year(local_inpa)*	*see a doctor locally with less serious diseases(less_serious_doctor)*
*imis*	0.0162**	0.0244***	0.0443*	0.00960	0.0212***	0.0550*	0.0190**	0.0246**	0.0351
	(0.00772)	(0.00874)	(0.0262)	(0.00633)	(0.00752)	(0.0316)	(0.00760)	(0.0110)	(0.0559)
*the need of hospitalization(need_inpa)*	√	√		√	√		√	√	
Other control variables	√	√	√	√	√	√	√	√	√
SES	√	√	√	√	√	√	√	√	√
Fixed effects of origin provinces	√	√	√	√	√	√	√	√	√
Fixed effects of flow-in cities	√	√	√	√	√	√	√	√	√
*N*	7404	7404	7404	6442	6442	6442	1626	1626	1626

*Note*: Robust standard errors are reported in parentheses. * *p* < 0.1, ** *p* < 0.05, *** *p* < 0.01. Other control variables include individual demographic characteristic, self-reported health status, having hypertension or diabetes, fitness time, having health examination or not in the past year. SES includes education, income, expenditure and immigration information. Same structure of dependent variable as in Table 2. The columns (1)– (3) have same structure as column (3) (6), and (8) in Table 2. The columns (4)– (6) have same structure as column (3) (6), and (8) in Table 3. The columns (7)– (9) have same structure as column (3) (6), and (8) in Table 4. In this regression, we drop the samples who joined the insurances type repeatedly

### Self-selection on *hukou*

As mentioned above, what kind of medical insurance residents enjoy depends on local policies and their *hukou* in China. Therefore, self-selection on IMIS does not exist. However, self-selection on *hukou* still exists. In general, those who can convert their or their families’ *hukou* from agricultural to non-agricultural have higher SES. For this reason, we repeat the previous benchmark regression process after dropping the individuals who have changed the nature of *hukou* (from agricultural to non-agricultural). The results are shown in Table 7, and the conclusion still has not changed. In fact, only 1.5% of *Migrants Parents* changed the nature of their *hukou* (from agricultural to non-agricultural) among the respondents in our data.

**Table 7:** Comparison of Health Care Utilization of Migrant Parents between IMISs and. URBMIs after Dropping Individuals Who Changed *hukou*

	(1)	(2)	(3)	(4)	(5)	(6)	(7)	(8)	(9)
	IMISs vs. non-IMISs			IMISs vs. NCMSs			IMISs vs. URBMIs		
	*hospitalized in the past year(inpa)*	*hospitalized locally in the past year(local_inpa)*	*see a doctor locally with less serious diseases(less_serious_doctor)*	*hospitalized in the past year(inpa)*	*hospitalized locally in the past year(local_inpa)*	*see a doctor locally with less serious(less_serious_doctor) diseases*	*hospitalized in the past year(inpa)*	*hospitalized locally in the past year(local_inpa)*	*see a doctor locally with less seriou(less_serious_doctor)s diseases*
*imis*	0.0173^**^	0.0238^***^	0.0448^*^	0.0115^*^	0.0199^***^	0.0493	0.0187^**^	0.0188^*^	0.0223
	(0.00778)	(0.00805)	(0.0248)	(0.00676)	(0.00751)	(0.0314)	(0.00724)	(0.0103)	(0.0522)
*the need of hospitalization(need_inpa)*	√	√		√	√		√	√	
Other control variables	√	√	√	√	√	√	√	√	√
SES	√	√	√	√	√	√	√	√	√
Fixed effects of origin provinces	√	√	√	√	√	√	√	√	√
Fixed effects of flow-in cities	√	√	√	√	√	√	√	√	√
*N*	7809	7809	7809	6804	6804	6804	1669	1669	1669

*Note*: Robust standard errors are reported in parentheses. * *p* < 0.1, ** *p* < 0.05, *** *p* < 0.01. Other control variables include individual demographic characteristic, self-reported health status, having hypertension or diabetes, fitness time, having health examination or not in the past year. SES includes education, income, expenditure and immigration information. Same structure of dependent variable as in Table 2. The columns (1)– (3) have same structure as column (3) (6), and (8) in Table 2. The columns (4)– (6) have same structure as column (3) (6), and (8) in Table 3. The columns (7)– (9) have same structure as column (3) (6), and (8) in Table 4. In this regression, we drop the the individuals who have changed the nature of *hukou*

### Exclude samples whose reason for migration is seeking medical treatment

As previously stated, *Migrants Parents* usually migrate “passively” because they move for their children, so there is little serious self-selection on mobility. However, suppose the reason for the older adults to migrate is to seek medical treatment. In that case, they will choose where it is easy to get medical treatment, which will result in serious self-selection on mobility and thus cause the confusion of regression conclusion. Fortunately, only 0.86% of *Migrants Parents* moved for medical treatment. After removing this part of the samples, the previous regression process is repeated, and the results are shown in Table 8. There is no difference between the results and benchmark results.

**Table 8:** Comparison of Health Care Utilization of Migrant Parents between IMISs and. URBMIs after Dropping the Individuals Who Moved for Medical Treatment

	(1)	(2)	(3)	(4)	(5)	(6)	(7)	(8)	(9)
	IMISs vs. non-IMISs			IMISs vs. NCMSs			IMISs vs. URBMIs		
	*hospitalized in the past year(inpa)*	*hospitalized locally in the past year(local_inpa)*	*see a doctor locally with less serious diseases(less_serious_doctor)*	*hospitalized in the past year(inpa)*	*hospitalized locally in the past year(local_inpa)*	*see a doctor locally with less serious diseases(less_serious_doctor)*	*hospitalized in the past year(inpa)*	*hospitalized locally in the past year(local_inpa)*	*see a doctor locally with less serious diseases(less_serious_doctor)*
*imis*	0.0151^**^	0.0216^***^	0.0412	0.0100	0.0187^**^	0.0439	0.0160^**^	0.0185^*^	0.0278
	(0.00736)	(0.00812)	(0.0252)	(0.00644)	(0.00733)	(0.0315)	(0.00673)	(0.0107)	(0.0529)
*the need of hospitalization(need_inpa)*	√	√		√	√		√	√	
Other control variables	√	√	√	√	√	√	√	√	√
SES	√	√	√	√	√	√	√	√	√
Fixed effects of origin provinces	√	√	√	√	√	√	√	√	√
Fixed effects of flow-in cities	√	√	√	√	√	√	√	√	√
*N*	7845	7845	7845	6827	6827	6827	1682	1682	1682

*Note*: Robust standard errors are reported in parentheses. * *p* < 0.1, ** *p* < 0.05, *** *p* < 0.01. Other control variables include individual demographic characteristic, self-reported health status, having hypertension or diabetes, fitness time, having health examination or not in the past year. SES includes education, income, expenditure and immigration information. Same structure of dependent variable as in Table 2. The columns (1)– (3) have same structure as column (3) (6), and (8) in Table 2. The columns (4)– (6) have same structure as column (3) (6), and (8) in Table 3. The columns (7)– (9) have same structure as column (3) (6), and (8) in Table 4. In this regression, we drop the *Migrants Parents* who moved for medical treatment

### Exclude samples whose YSM is less than one

As we mentioned, the positive self-selection of migrants posits that only the healthiest and most motivated individuals choose to move to a new place, while less healthy and weaker individuals stay behind. Considering the *Migrant Parents* might be healthier than the native population when they first arrived in the host cities, we dropped the samples with YSM less than one and repeated previous regression. The results shown in Table 9 reveals that there is no difference between the results and benchmark results.

**Table 9:** Comparison of Health Care Utilization of Migrant Parents between IMISs and. URBMIs after Dropping the Individuals Whose YSM is Less Than One

	(1)	(2)	(3)	(4)	(5)	(6)	(7)	(8)	(9)
	IMISs vs. non-IMISs			IMISs vs. NCMSs			IMISs vs. URBMIs		
	*hospitalized in the past year(inpa)*	*hospitalized locally in the past year(local_inpa)*	*see a doctor locally with less serious diseases(less_serious_doctor)*	*hospitalized in the past year(inpa)*	*hospitalized locally in the past year(local_inpa)*	*see a doctor locally with less serious diseases(less_serious_doctor)*	*hospitalized in the past year(inpa)*	*hospitalized locally in the past year(local_inpa)*	*see a doctor locally with less serious diseases(less_serious_doctor)*
*imis*	0.0164*	0.0212**	0.0354	0.00996	0.0169**	0.0454	0.0188**	0.0202*	0.0229
	(0.00906)	(0.00856)	(0.0267)	(0.00796)	(0.00745)	(0.0320)	(0.00751)	(0.0113)	(0.0554)
*the need of hospitalization(need_inpa)*	√	√		√	√		√	√	
Other control variables	√	√	√	√	√	√	√	√	√
SES	√	√	√	√	√	√	√	√	√
Fixed effects of origin provinces	√	√	√	√	√	√	√	√	√
Fixed effects of flow-in cities	√	√	√	√	√	√	√	√	√
*N*	7325	7325	7325	6348	6348	6348	1641	1641	1641

*Note*: Robust standard errors are reported in parentheses. * *p* < 0.1, ** *p* < 0.05, *** *p* < 0.01. Other control variables include individual demographic characteristic, self-reported health status, having hypertension or diabetes, fitness time, having health examination or not in the past year. SES includes education, income, expenditure and immigration information. Same structure of dependent variable as in Table 2. The columns (1)– (3) have same structure as column (3) (6), and (8) in Table 2. The columns (4)– (6) have same structure as column (3) (6), and (8) in Table 3. The columns (7)– (9) have same structure as column (3) (6), and (8) in Table 4. In this regression, we drop the individuals whose YSM is less than one

## Potential mechanisms

To investigate the mechanisms of IMIS relieving the difficulty of medical treatment in migrant destinations, we should discuss the reason for IMIS improving the willingness of the *Migrants Parents* to be hospitalized in the destination. In this paper, we can only preliminarily investigate the causes for IMIS improving hospitalization intention and give our suggestive evidence through simple descriptive statistics because of limited observations.

In the samples used in this paper, 146 people got illnesses that doctors thought required hospitalization, but they give up. 10 of them are IMISs, and 136 are Non-IMISs. It, of course, once again proves that IMIS has greatly reduced the possibility of *Migrants Parents* being sick but not going to treatment. For those responders who did not be hospitalized, the questionnaire further inquired why they choose not to be hospitalized. The statistical results are shown in Table 8. There are two important results: first, 15 people (11.03%) in the Non-IMISs group chose not to be hospitalized because of “inconvenient reimbursement.” In contrast, 0 people complained about “inconvenient reimbursement” in the IMISs group. Therefore, it indicates that IMIS might have improved the willingness to be hospitalized in the migrant destination, probably because IMIS has improved the convenience of medical expense reimbursement. Second, 38 people (27.94%) in Non-IMISs chose not to be hospitalized because of “poor.” In contrast, only one person (10%) in IMISs chose “poor,” which indicates that another mechanism for IMIS to improve the intention of hospitalization in the destination might be relieving the economic constrain caused by medical expenses to increase the health insurance benefits. Based on the above discussion, it might be concluded that IMIS can alleviate the difficulty of seeking medical care in migrant destinations mainly through two ways: improving the convenience of medical expense reimbursement and enhancing health insurance benefits. Fortunately, both enhancing the convenience of medical expense reimbursement and improving health insurance benefits are goals and original intentions of IMIS.

## Conclusion and discussion

This paper discusses the influence of IMIS in China on the difficulty of migrant parents to seek medical treatment in a migrant destination. We find that IMIS indeed alleviates the problem of aging *Migrants Parents* in seeking medical treatment in a migrant destination. It can be reflected that IMISs are more likely to choose hospitalization and seek medical treatment in the migrant destination than Non-IMISs. In order to reduce the possible interference of selection bias on the conclusion, we further subdivide Non-IMISs into NCMSs and URBMIs, which are respectively compared with IMISs. It has proved that the conclusion of IMIS alleviating the difficulty of getting medical treatment in migrant destinations still remains.

Our paper attempts to discuss further the channels of IMIS easing medical treatment difficulty in the migrant destination. The result gives us good inspiration: IMIS can alleviate the problem of seeking medical care in migrant destinations mainly through two ways: improving the convenience of medical expense reimbursement and relieving the economic constrain. Therefore, it has been found in the survey that compared with Non-IMISs, almost no IMISs give up hospitalization in migrant destinations because of inconvenient reimbursement and economic constrain. However, before piloting the IMIS, most NCMS schemes require prior approval for the use of services in non-local facilities, and the process tends to be somewhat lengthy, thus creating an additional barrier for the *Migrants Parents* to use health care services locally [12, 63, 64]. In this case, even though some NCMS schemes covered out-of-county bills, the reimbursement ratio tends to be lower, while outpatient costs are typically non-reimbursable [52].

What we investigate in this study is of significant policy implication. This paper provides empirical evidence for China’s adherence to the IMIS reform direction. Even China has accomplished a high health insurance coverage rate, 99.36% [65], the vulnerable groups, such as older migrants, are still in the disadvantaged position in terms of the limited access to health care and insufficient health care utilization. Relative to the WHO 2010 World Health Report, it is proposed that a country moving towards universal coverage should consider three dimensions: the population (who is covered), the services (which services are included), and the costs (proportion of the costs that is covered). China’s IMIS reform has effectively promoted the equity of services packages and healthcare costs between groups [3, 66, 67]. Although IMIS reform involves many aspects of the interests’ redistribution, in which many parts of medical departments may need to pay any costs, it is worthwhile to pay such a price from the conclusion of this study. IMIS can ensure that when *Migrants Parents* need to be hospitalized, they will accept hospitalization and choose to go to the hospital for medical treatment in the migrant destination. They will no longer give up hospitalization or go back to their hometown for medical treatment because of the complicated reimbursement procedures.

Our study of IMIS also sheds light on achieving universal health coverage and healthcare reform for the world. As China has already accomplished universal health coverage in the population dimension, building IMIS aims to expand the services package and enhance financial protection ability for everyone health insurance beneficiaries at a higher level. It is consistent with the United Nations Sustainable Development Goals in 2016, which committed countries to achieve universal health coverage by 2030, focusing on essential health services and financial protection [68]. Our results are consistent with the research in other countries that built the integrated health insurance system, especially those with large population disparity regarding social-economic status as China. For example, in Ghana, Kenya, and Thailand, the national health insurance schemes could increase the health care utilization of poor and vulnerable groups [69–71]. In terms of the United States, the country with the highest health expenditure globally, the fragmentation of the healthcare system causes complicated insurance relationships, inadequate preventive care, and increased administrative cost. The financing of different healthcare sectors in the United States is distributed across various distinct and often competing entities, each with its objectives, obligations, and capabilities, which also affect the efficiency and the quality of health care [72]. Prior researches based on the US insurance marketplace suggests that concentration and utilization are positively related [73–75], which is consistent. In addition, some studies show that the concentration of insurance companies does negotiate lower hospital prices [73, 76, 77]. As insurers consolidate, hospitals may increasingly view quality as a means to maintain bargaining leverage in their negotiations [75].

Several limitations of this study must be noted. First, the conclusions of our study are primarily descriptive and illustrative and do not represent canonical causal effects. Secondly, due to data limitations, especially the small sample of responders whose doctors think need hospitalization does not choose hospitalization, it is impossible to conduct a more detailed empirical analysis. Meanwhile, we also cannot control the level of the hospitals of hospitalization. But discussion of the work to give us suggestive evidence is more confined to the descriptive statistical analysis. Those limitations motive us to address these shortcomings in our future research.

## Data Availability

The data is available on the website of China Migrants Dynamic Survey (https://chinaldrk.org.cn/wjw/#/home). The data belongs to the China National Health Commission and is free for researchers. We submitted the application in the website and received the data package.

## Abbreviations

IMIS: Integrated Medical Insurance System
NCMS: New Rural Cooperative Medical Scheme
URBMI: Urban Resident Basic Medical Insurance
UEBMI: Urban Employee Basic Medical Insurance
CMDS: China Migrants Dynamic Survey

## Footnotes

1 “Hukou” is a system of household registration used in mainland China. A household registration record officially identifies a person as a permanent resident of an area and includes identifying information such as name, parents, spouse and date of birth.

2 Some of the provinces have different pooling and organization of the health insurance schemes. For example in Ningxia, NCMS are pooled at provincial level, while many URBMI and UEBMI are managed at country level of Zhejiang, Jiangsu, etc.

3 China Migrants Dynamic Survey, https://www.chinaldrk.org.cn/

4 We use OLS for regression in this paper. Since the dependent variable is 0–1, it is equivalent to using the linear probability model. We use OLS because its form is simpler and its coefficients are more intuitive. We also tried the Probit model and the conclusions did not change.
