# Correction to: Does integrated medical insurance system alleviate the difficulty of using cross-region health care for the migrant parents in China-- evidence from the China migrants dynamic survey

**DOI:** 10.1186/s12913-021-07305-3

**Published:** 2021-12-02

**Authors:** Chao Ma, Shutong Huo, Hao Chen

**Affiliations:** 1 Southeast University, School of Economics and Management, Nanjing, Jiangsu China; 2 University of California, Program of Public Health, Irvine, CA USA; 3 University of International Business and Economics, Institute of International Economy, Beijing, China

**Correction to: BMC Health Serv Res 21, 1053 (2021)**

**https://doi.org/10.1186/s12913-021-07069-w**

Following publication of the original article [1], an error was identified in the article title. The first letter of the word “Care” should not be capitalized.

The article title should read, and the change has been highlighted in **bold typeface**.

Does integrated medical insurance system alleviate the difficulty of using cross-region health **care** for the Migrant Parents in China-- evidence from the China migrants dynamic survey.

In addition, the city and country of the author affiliation 3 are incorrect. The updated author affiliation 3 should be:

University of International Business and Economics, Institute of International Economy, Beijing, China.

The original article [1] has been corrected.
